# Effectiveness of Cardiopulmonary Exercise Testing as an Incentive to Enhance Outpatient Cardiac Rehabilitation Participation in Acute Coronary Syndrome Survivors: A Study Protocol for a Randomized Controlled Trial with Determinant Analysis

**DOI:** 10.3390/jcm15010319

**Published:** 2026-01-01

**Authors:** Yuchun Lee, Chin-Yin Huang, Hungchin Ho, Yuan-Yang Cheng

**Affiliations:** 1 Department of Physical Medicine and Rehabilitation, Taichung Veterans General Hospital, Taichung 407219, Taiwan; 2 Department of Industrial Engineering and Enterprise Information, Tunghai University, Taichung 407224, Taiwan; 3 Cardiovascular Center, Taichung Veterans General Hospital, Taichung 407219, Taiwan

**Keywords:** acute coronary syndrome, cardiac rehabilitation, cardiopulmonary exercise testing

## Abstract

**Background**: Despite clear evidence supporting the benefits of outpatient cardiac rehabilitation for acute coronary syndrome survivors, participation rates remain low. Many patients face person-level and system-level barriers for outpatient rehabilitation, and their motivation often wanes soon after discharge. Cardiopulmonary exercise testing provides individualized physiological information and may act as an external cue that enhances engagement, yet no randomized trial has evaluated whether announcing a scheduled test can influence rehabilitation attendance. **Methods**: This single-center, parallel-group, single-blind randomized controlled trial investigates whether informing patients during hospitalization that a cardiopulmonary exercise test will be conducted at their first rehabilitation visit increases early outpatient attendance. Patients with acute coronary syndrome are randomized 1:1 to receive either standard discharge education or the same education plus an exercise testing announcement. All other clinical care follows routine practice. The primary endpoint is completion of the first rehabilitation clinic visit within 30 days. Secondary outcomes include attending at least six rehabilitation sessions within 12 weeks, actual exercise testing completion, and any safety events. The analyses will follow the intention-to-treat principle and will use logistic regression and time-to-event models. The planned sample size is 200 participants.

## 1. Introduction

### 1.1. The Crucial Role of Cardiac Rehabilitation and the Implementation Gap

Cardiovascular diseases remain the leading global cause of death, accounting for more than 19 million deaths in 2022 [1]. Acute coronary syndrome (ACS) arises from the sudden disruption of an atherosclerotic plaque and may result in myocardial infarction or unstable angina [2]. Although advances in percutaneous coronary intervention and medical therapy have markedly improved short-term survival [3], many patients continue to experience limited long-term functional recovery and impaired quality of life [4].

Cardiac rehabilitation is a comprehensive secondary prevention intervention that includes exercise training, risk factor modification, and psychosocial support. Strong evidence from randomized trials and meta-analyses have shown that receiving cardiac rehabilitation reduces cardiovascular mortality, all-cause mortality, and re-hospitalization, along with improvements in exercise capacity and quality of life among ACS patients [5,6]. Guidelines from the American Heart Association (AHA), the American College of Cardiology (ACC), and the European Society of Cardiology (ESC) have recommended cardiac rehabilitation as a Class I intervention for eligible patients [7,8].

### 1.2. The Implementation Gap of Cardiac Rehabilitation

Despite this strong evidence, cardiac rehabilitation participation rates remain low. In many regions of the world, less than 20–30% of eligible patients attend outpatient cardiac rehabilitation, with even lower rates reported in parts of Asia [9,10,11]. In Taiwan, the actual completion rates of outpatient cardiac rehabilitation have been reported to be below 10% [12,13].

This persistent gap between proven efficacy and real-world practice raises important questions. Prior studies have proposed several patient-level obstacles, including insufficient understanding of cardiac rehabilitation, work or family obligations, transportation burden, and low perceived need for structured exercise [14,15]. System-level gaps such as inconsistent referral processes, lack of automatic referral pathways and staffing, and limited program capacity further contribute to low participation [16,17]. Psychosocial factors, including inadequate family support, anxiety, and persistent uncertainty following acute coronary events, also influence engagement, particularly among women and older adults [18,19].

Increasing attention has been directed toward alternative and non-traditional models of cardiac rehabilitation delivery, with recent literature emphasizing the importance of comprehensive and personalized approaches to address logistical, occupational, and motivational barriers associated with conventional rehabilitation programs [20,21]. Successful examples of cardiac rehabilitation implementation have been reported in highly structured clinical settings, where multidisciplinary teams and well-established referral and follow-up pathways enable high levels of patient engagement and program completion [22]. Automatic referral with liaison follow-up, nurse-led case management, structured education, financial incentives, mobile health applications, and text-messaging programs have each demonstrated varying degrees of success in promoting rehabilitation engagement [16,23,24,25,26,27]. However, many of these interventions depend on additional staffing, long-term funding, or specialized digital platforms, which limit widespread scaling, particularly in settings with constrained resources or low digital literacy [17,28,29]. Given these challenges, simple and universally applicable behavioral approaches that can be integrated into routine workflows are needed.

### 1.3. The Role and Clinical Importance of CPET

Cardiopulmonary exercise testing (CPET) is widely used in cardiovascular and sports medicine to assess functional capacity, identify exercise intolerance, and guide exercise prescription. It provides objective, individualized metrics such as peak oxygen uptake (peak VO_2_) and ventilatory efficiency (VE), which are strongly associated with prognosis in coronary artery disease and reflects the recovery of cardiopulmonary fitness [30,31]. Contemporary guidelines consistently endorse CPET as a valuable tool for risk stratification and for tailoring exercise training in cardiac rehabilitation programs [7,32]. Despite its central role in clinical evaluation, CPET has traditionally been used primarily for diagnostic clarification, risk assessment, and exercise prescription. Its function has rarely been considered beyond the physiological or diagnostic domain, and its potential influence on patient motivation or behavior has remained unexplored.

### 1.4. Behavioral Potential of CPET: Opportunities from a Health Psychology Perspective

Although CPET has been viewed mainly as a physiological test, theoretical perspectives from behavioral science suggest that its announcement and framing could have motivational value. The Health Information Seeking Behavior (HISB) model indicates that individuals who have experienced health uncertainty or vulnerability actively seek personalized health information, particularly when they expect actionable feedback [33,34]. For patients recovering from ACS, the prospect of receiving objective CPET results might trigger this information-seeking drive.

Similarly, the concept of a “teachable moment” suggests that acute health events temporarily heighten attention to risk and openness to change [35,36]. However, this heightened motivation declines soon after discharge. Announcing a forthcoming CPET may help to extend this motivational window by creating a personalized event linked directly to their recovery.

The Health Belief Model (HBM) further supports the possibility that CPET announcement may shape behavior. By emphasizing the need to evaluate cardiopulmonary function shortly after hospitalization, the announcement may enhance perceived susceptibility, reinforce the severity of the illness experience, highlight the benefit of rehabilitation for improving CPET outcomes, and act as a direct “cue to action” [37,38]. Together, these frameworks suggest that when framed deliberately, CPET could serve not only as a physiological assessment but also as a behavioral trigger.

### 1.5. Integrating Clinical and Behavioral Perspectives: Study Hypothesis

Building on these insights, the present study examines the potential of CPET beyond its conventional diagnostic role. We hypothesize that patients who are clearly informed about an upcoming CPET may exhibit stronger motivation to return for early outpatient rehabilitation. The trial is therefore designed to examine whether embedding this cue within routine discharge planning can meaningfully influence initial attendance and serve as an accessible, scalable strategy to address the persistent gap between guideline-recommended cardiac rehabilitation and actual participation.

## 2. Materials and Methods

### 2.1. Study Design and Setting

This is a prospective, parallel-group, single-blind randomized controlled trial conducted at Taichung Veterans General Hospital (TCVGH), a tertiary medical center in Taiwan. The study was approved by the Institutional Review Board (IRB) of TCVGH (CE22225A) on 25 May 2022, and registered at ClinicalTrials.gov (NCT05401240) on 29 May 2022. Eligible patients were identified during hospitalization for ACS. Screening, informed consent, and random allocation were completed before discharge. All clinical care followed contemporary ACS and chronic coronary disease guidelines [7,8,13]. The study intervention did not restrict or delay any indicated diagnostic tests or treatments.

**Figure 1:**
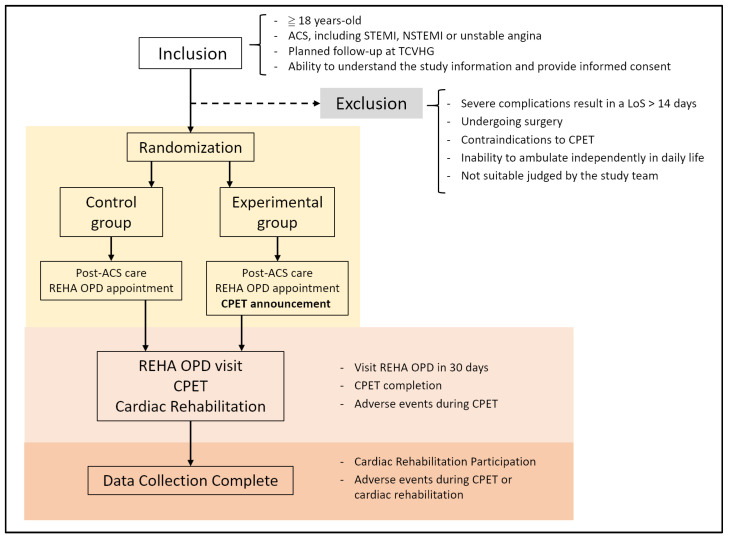
Study scheme.

The trial protocol was developed in line with the SPIRIT 2025 recommendations for randomized trial protocols [39] and the conduct and reporting of this study will follow CONSORT guidelines for randomized controlled trials. Patient recruitment began in September 2023 at TCVGH and is currently ongoing. The schematic enrollment, interventions, and assessments flowchart is demonstrated in Figure 1.

### 2.2. Participants

Patients are eligible if they meet all of the following criteria:

1. Aged at least 18 years.
2. Hospitalization with a primary diagnosis of ACS, including S-T elevated myocardial infarction (STEMI), non-ST elevated myocardial infarction (NSTEMI), or unstable angina, managed with standard evidence-based therapy.
3. Planned post-discharge follow-up at the cardiology outpatient department (OPD) of TCVGH.
4. Able to understand the study information and provide written informed consent.

Patients are excluded if they meet any of the following criteria:

1. Severe medical complications during hospitalization resulting in a length of stay (LoS) longer than 14 days, such as respiratory failure or acute kidney injury requiring intensive treatment.
2. Undergoing open-heart surgery during the index hospitalization.
3. Contraindications to CPET according to contemporary standards [31].
4. Severe functional dependence with inability to ambulate independently in daily life due to neurological or musculoskeletal conditions.
5. Any other condition judged by the clinical team to make participation unsafe or infeasible.

### 2.3. Interventions

All participants will receive standardized in-hospital education delivered by trained staff. The education covers the goals and components of cardiac rehabilitation, the benefits of regular physical activity, basic home exercise recommendations, medication adherence, and risk factor management. Before discharge, the research team will schedule a first visit at the rehabilitation clinic within approximately 30 days after discharge for all enrolled patients, and this appointment will be documented in the discharge summary. The selection of attendance within 30 days of hospital discharge is based on evidence that earlier scheduling within the first few weeks is associated with higher initial participation, while scheduling beyond one month is linked to lower uptake [40,41].

Patients in the CPET announcement group (intervention arm) will receive the standard education supplemented by a specific behavioral strategy. The research team will formally announce that a CPET would be arranged at their first rehabilitation outpatient visit. The educator uses a standardized script to emphasize that CPET would assess their current cardiopulmonary fitness, compare it with age-matched norms, and guide the intensity of subsequent exercise training. Patients are given a one-page leaflet describing the CPET procedure, safety measures, and clinical benefits in simple language. The discharge instructions explicitly note “rehabilitation clinic visit and cardiopulmonary exercise testing” to reinforce the expectation.

In the standard care group (control arm), patients will receive the same standard education and pre-scheduled rehabilitation clinic appointment, but CPET will not be proactively mentioned during hospitalization.

Ethically, the intervention is defined strictly as the “pre-discharge declaration” and the provision of informational materials. Access to clinical services remains equitable; physicians in both arms retain full discretion to order CPET based on clinical indications post-discharge.

### 2.4. Randomization, Allocation Concealment and Blinding

A computer-generated random sequence with a 1:1 allocation ratio will be prepared by a statistician not involved in recruitment or outcome assessment. Sequentially numbered, opaque, sealed envelopes containing group assignments are used to ensure allocation concealment. After an eligible patient provides written informed consent and baseline data are collected, the educator opens the next envelope to reveal the assignment and deliver the appropriate intervention.

Outcome assessors and data analysts are blinded to group allocation. Complete blinding of outpatient clinicians is not feasible because some patients in the intervention group might spontaneously mention the planned CPET during follow-up, but clinicians are not informed of group assignment by the study team and are instructed not to seek this information.

### 2.5. Outcome and Covariates

The primary outcome is their attendance at the rehabilitation clinic within 30 days of hospital discharge. Attendance is defined as completion of at least one scheduled rehabilitation outpatient visit during this period at TCVGH. Attendance is verified via the hospital’s electric medical record system. Given the study’s focus on institutional workflow integration, visits to external facilities are not counted toward the primary endpoint to maintain data consistency.

Secondary outcomes include the following:

1. Cardiac rehabilitation program participation: completion of a predefined early rehabilitation course within 12 weeks after discharge, defined as attending at least six supervised rehabilitation sessions.
2. CPET completion: whether a CPET is ultimately performed during follow-up, irrespective of group.
3. Safety: occurrence of adverse events potentially related to exercise or rehabilitation during the observation period, such as syncope during exercise sessions, sustained arrhythmias requiring urgent management, or emergency department visits or rehospitalization that related to therapeutic exercise

We will also collect the following covariates and potential determinants:

1. Demographic factors—age, sex, body mass index (BMI).
2. Socioeconomic and lifestyle factors—education level, employment and physical workload, physical activity level prior to ACS, and current smoking status.
3. Social context—residential distance from the hospital and whether a family member accompanied the patient during hospitalization.
4. Comorbidities—hypertension, diabetes mellitus, dyslipidemia, and renal impairment.
5. ACS characteristics—ACS type based on electrocardiographic (ECG) findings (STEMI or NSTEMI), peak creatine kinase (CK) level, left ventricular ejection fraction (LVEF) by ECG, and hospitalization LoS.

In addition, to assess key behavioral constructs referenced in the theoretical framework, cardiac self-efficacy will be measured at baseline using the validated Cardiac Self-Efficacy Scale (CSES). The CSES is a disease-specific instrument designed to evaluate patients’ confidence in managing symptoms, maintaining physical function, and engaging in recommended health behaviors following cardiac events [42]. The questionnaire will be administered at the time of hospital discharge, prior to randomization. Higher scores indicate greater perceived self-efficacy related to cardiac disease management.

### 2.6. Sample Size Calculation

The sample size was estimated for the primary outcome of 30-day rehabilitation clinic attendance. Based on local and regional data indicating very low CR participation (<10%) after ACS [9,11,12,13]. Prior evidence demonstrates that low-cost, scalable interventions incorporating structured information delivery or “cue-to-action” strategies can produce absolute increases in cardiac rehabilitation participation of approximately 14–20% [43,44]. Therefore, we hypothesized that announcing a forthcoming CPET could increase this rate to 25%, corresponding to a 15% absolute improvement which is considered a conservative and clinically meaningful effect size, representing the minimum improvement likely to justify changes to standard discharge education and follow-up pathways. With a two-sided alpha of 0.05 and 80% power, this required approximately 100 patients per group (total 200). To allow for up to 10% attrition rate, we aimed to enroll at least 220 patients.

### 2.7. Statistical Analysis

All analyses will follow the intention-to-treat principle, and all randomized patients will be included in the primary analysis. Continuous variables are presented as means and standard deviations, and categorical variables as counts and percentages. Baseline characteristics are compared between groups using independent-samples *t* tests for continuous variables and chi-square tests for categorical variables.

For the primary outcome, participants who are lost to follow-up or whose attendance at the rehabilitation clinic cannot be confirmed through the electronic medical record system within the predefined 30-day window will be classified as non-attendees. The proportion of attendees between groups will be compared using the chi-square test, with crude odds ratios and 95% confidence intervals reported. A multivariable logistic regression model will then be fitted to estimate adjusted odds ratios (AORs) with 95% confidence interval for the effect of the intervention on attendance, adjusting for a prespecified set of clinically relevant covariates, including age, sex, ACS type (STEMI vs. NSTEMI), residential distance from the hospital, and baseline physical activity level, as these variables have been linked to cardiac rehabilitation referral/enrolment patterns or post-discharge health behaviors in patients with ACS [15,45,46].

In addition, a broader set of demographic, socioeconomic, clinical, and disease-related variables will be examined in secondary and exploratory analyses to further characterize factors associated with rehabilitation attendance: BMI; education level; employment and physical workload; smoking status; family accompaniment during hospitalization; comorbidities (hypertension, diabetes mellitus, dyslipidemia, and renal impairment); ACS-related characteristics, including peak CK level, LVEF, and LoS; and CSES score. Where appropriate, effect modification by key patient and clinical characteristics will be explored by including interaction terms between the intervention and selected variables.

Missing data in baseline covariates will be assessed for patterns and mechanisms. If the proportion of missing data is substantial, multiple imputation using chained equations will be applied as a sensitivity analysis, and results will be compared with complete-case analyses. All tests will be two-sided with a significance level of 0.05. Statistical analyses will be performed using SPSS (Version 29, IBM Corp., Armonk, NY, USA).

### 2.8. Oversight and Monitoring

Because this study involves a minimal-risk, non-pharmacological behavioral intervention that does not alter standard clinical care or expose participants to additional medical procedures, a formal independent Data Monitoring Committee (DMC) is not required. No investigational products, device interventions, or invasive procedures are involved, and all participants receive standard clinical management irrespective of trial allocation.

Adverse events related to exercise testing or rehabilitation, including but not limited to exercise-induced syncope, clinically significant arrhythmia, or other unexpected medical events, will be captured through the electronic medical record system during follow-up visits. Such events will be identified and reported by the attending rehabilitation physician responsible for conducting the cardiopulmonary exercise testing or supervising rehabilitation sessions. The principal investigator will review all safety data at monthly intervals. Any serious adverse events will be reported to the IRB of TCVGH within 24 h of identification.

Study oversight is performed by the principal investigator and the institutional research office at TCVGH, which ensures compliance with ethical standards and trial governance requirements. No interim analyses or stopping guidelines are planned, as the intervention is informational in nature and does not introduce clinical risk. Trial monitoring focuses on data accuracy and timely reporting of any unexpected issues. Research staff will review screening logs, enrollment flow, and data quality at regular intervals, and any concerns will be documented and addressed according to institutional procedures. Given the low-risk profile and the absence of safety-related endpoints, no external monitoring body is deemed necessary.

No interim analyses or formal stopping guidelines are planned, as the intervention is informational in nature and does not introduce additional clinical risk. Trial monitoring focuses on data completeness, accuracy, and protocol adherence. Research staff will conduct regular internal reviews of screening logs, enrollment flow, and data quality, and any protocol deviations or unexpected issues will be documented and managed according to institutional procedures. Given the low-risk profile of the study and the absence of safety-related endpoints, no external monitoring body is considered necessary.

### 2.9. Confidentiality

All participant information will be collected, stored, and managed in accordance with institutional policies and applicable privacy regulations. Identifiable data (including name, medical record number, and contact information) will be stored separately from study datasets in a secure, password-protected digital repository accessible only to authorized study personnel. Research data used for analysis will be de-identified and assigned a unique study code. No identifiable information will be shared externally, included in publications, or transferred outside the institution. During follow-up and data entry, paper documents will be kept in locked research cabinets within restricted-access areas. After study completion, de-identified datasets will be retained for the period required by institutional guidelines, while identifiable information will be destroyed to ensure long-term confidentiality.

## 3. Discussion

### 3.1. Rationale and Innovation

This trial represents the first randomized evaluation of repurposing a physiological diagnostic tool as a behavioral “nudge”. While the prognostic utility of CPET is well-documented, this protocol innovates by leveraging the anticipation of the test itself to drive patient engagement. This protocol innovates by integrating clinical diagnostics with health psychology theories. By framing CPET as a “teachable moment” and a “cue to action”, we aim to transform the diagnostic process itself into a motivational lever. If effective, this strategy addresses the critical implementation gap in cardiac rehabilitation without requiring expensive digital infrastructure or labor-intensive case management.

### 3.2. Strength and Limitation

This study has several strengths. First, the randomized design minimizes selection bias, providing high-quality evidence regarding the causal effect of the announcement strategy. Furthermore, the intervention is low-cost and highly scalable, utilizing existing hospital resources rather than external funding or technology. At last, the inclusion of a determinant analysis allows for understanding of “who” responds to this incentive, aiding in the future personalization of care.

However, limitations are anticipated. As a single-center study, the generalizability of findings to rural hospitals or different healthcare systems may be limited. Additionally, due to the nature of the behavioral intervention, blinding of participants is not possible, which may introduce performance bias. Finally, the primary endpoint focuses solely on initial attendance; consequently, the study design does not enable the assessment of long-term adherence or hard clinical outcomes, such as re-hospitalization or mortality, despite existing evidence demonstrating favorable long-term outcomes with sustained participation in cardiac rehabilitation [21].

### 3.3. Clinical Implication

The results of this trial could have immediate implications for clinical practice. If the hypothesis is confirmed, incorporating a “CPET announcement” into standard discharge pathways could become a routine, evidence-based strategy to boost rehabilitation engagement. Conversely, if the intervention proves ineffective, the determinant analysis will still provide valuable data on the barriers facing Taiwanese ACS patients, guiding the development of alternative strategies.

## 4. Conclusions

Improving participation in cardiac rehabilitation is a global public health priority. This protocol describes a novel, theory-driven approach to bridge the gap between acute care and outpatient rehabilitation. By evaluating whether a scheduled clinical test can act as a behavioral nudge, this study seeks to provide a practical solution to enhance the continuum of care for ACS survivors.

## Data Availability

Data sharing is not applicable to this article as no datasets were generated or analyzed during the current study protocol phase. The final dataset will be available from the corresponding author on reasonable request upon completion of the trial.
